# Sialic Acid-Like Sugars in Archaea: Legionaminic Acid Biosynthesis in the Halophile *Halorubrum* sp. PV6

**DOI:** 10.3389/fmicb.2018.02133

**Published:** 2018-09-07

**Authors:** Marianna Zaretsky, Elina Roine, Jerry Eichler

**Affiliations:** ^1^Department of Life Sciences, Ben-Gurion University of the Negev, Beersheva, Israel; ^2^Molecular and Integrative Biosciences Research Programme, University of Helsinki, Helsinki, Finland

**Keywords:** archaea, halophile, *Halorubrum*, legionaminic acid, *N*-formylation, N-glycosylation, S-layer glycoprotein

## Abstract

N-glycosylation is a post-translational modification that occurs in all three domains. In Archaea, however, N-linked glycans present a degree of compositional diversity not observed in either Eukarya or Bacteria. As such, it is surprising that nonulosonic acids (NulOs), nine-carbon sugars that include sialic acids, pseudaminic acids, and legionaminic acids, are routinely detected as components of protein-linked glycans in Eukarya and Bacteria but not in Archaea. In the following, we report that the N-linked glycan attached to the S-layer glycoprotein of the haloarchaea *Halorubrum* sp. PV6 includes an *N*-formylated legionaminic acid. Analysis of the *Halorubrum* sp. PV6 genome led to the identification of sequences predicted to comprise the legionaminic acid biosynthesis pathway. The transcription of pathway genes was confirmed, as was the co-transcription of several of these genes. In addition, the activities of LegI, which catalyzes the condensation of 2,4-di-*N*-acetyl-6-deoxymannose and phosphoenolpyruvate to generate legionaminic acid, and LegF, which catalyzes the addition of cytidine monophosphate (CMP) to legionaminic acid, both heterologously expressed in *Haloferax volcanii*, were demonstrated. Further genome analysis predicts that the genes encoding enzymes of the legionaminic acid biosynthetic pathway are clustered together with sequences seemingly encoding components of the N-glycosylation pathway in this organism. In defining the first example of a legionaminic acid biosynthesis pathway in Archaea, the findings reported here expand our insight into archaeal N-glycosylation, an almost universal post-translational modification in this domain of life.

## Introduction

Post-translational modifications represent a major source of proteomic expansion. Of the numerous processing events that can modify a protein, N-glycosylation, or the covalent attachment of a glycan to selected Asn residues of a target protein, is the most complex. Long held to be specific to Eukarya, it is now clear that Archaea and Bacteria also perform this post-translational modification. However, while N-glycosylation in Bacteria is believed to solely occur in the delta/epsilon proteobacteria (Nothaft and Szymanski, 2010), such protein processing appears to be an almost universal trait in Archaea (Kaminski et al., 2013a). As insight into N-glycosylation across evolution accumulates, unique aspects of this post-translational modification in each domain are being defined (Jones et al., 2009; Maita et al., 2010; Larkin and Imperiali, 2011; Koomey and Eichler, 2017; Eichler and Imperiali, 2018). For instance, domain-related diversity in the sugar composition of N-linked glycans serves to distinguish eukaryal, bacterial, and archaeal N-glycosylation (Schwarz and Aebi, 2011; Eichler, 2013).

In Eukarya, N-glycosylation begins in the endoplasmic reticulum (ER) and is completed in the Golgi. Whereas glycoproteins leaving the ER bear a common N-linked glycan core (Aebi, 2013), considerable diversity is introduced at the level of the Golgi, largely through the addition of any number of a variety of sialic acids (Angata and Varki, 2002; Chen and Varki, 2010; Cohen and Varki, 2010; Deng et al., 2013). Sialic acids correspond to a large group (>50 members) of structurally distinct molecules that are part of a larger group of nine-carbon sugars termed nonulosonic acid (NulO) sugars (Varki et al., 2017) and which are important for a variety of eukaryal process, including development, recognition, and immune responses (Varki and Gagneux, 2012; Schnaar et al., 2014; Stencel-Baerenwald et al., 2014; Pearce and Läubli, 2016). Although less common, some Bacteria also present sialic acids, either synthesized *de novo* or scavenged from other cells, as components of cell-surface structures (Vimr et al., 2004; Ferrero and Aparicio, 2010; Vimr, 2013). Indeed, genomic analysis predicts sialic acid biosynthetic pathways in a variety of bacterial groups (Lewis et al., 2009). Bacteria can also synthesize saccharides assigned to two other NulO sugar families, pseudaminic acids, and legionaminic acids, for use in protein glycosylation (Knirel et al., 2003; Vimr et al., 2004; Schoenhofen et al., 2006; McNally et al., 2007; Glaze et al., 2008; Lewis et al., 2009; Morrison and Imperiali, 2014). First identified in the O-polysaccharide of lipopolysaccharide in *Legionella pneumophila*, the cause of Legionnaires’ disease (Knirel et al., 1994), legionaminic acid (5,7-diacetamido-3,5,7,9-tetradeoxy-D-glycero-D-galacto-NulO) is thought to serve as a molecular mimic of sialic acid, allowing the pathogenic bacteria that synthesize this sugar to evade detection by the immune system of infected eukaryal hosts (Schoenhofen et al., 2009).

Given how the N-linked glycans decorating archaeal glycoproteins present a degree of diversity in sugar composition not found elsewhere (Schwarz and Aebi, 2011; Eichler, 2013; Jarrell et al., 2014), it is surprising that so few Archaea encode enzymes predicted to participate in NulO sugar biogenesis pathways. In surveying 122 archaeal genomes, putative components of sialic acid, pseudaminic acid, or legionaminic acid biosynthesis pathways were only predicted in less than 20% of the cases considered (Kandiba and Eichler, 2013). Indeed, genes putatively encoding complete pathways were only detected in six species. Even less evidence has been offered for any NulO as being associated with an archaeal glycoprotein. Pseudaminic acid was detected in an extract of *Methanobrevibacter smithii* (Lewis et al., 2009), a methanogen found in the human gut microbiome (Miller et al., 1982), correcting an earlier report of sialic acid being present in this organism (Samuel et al., 2007). This study did not, however, determine whether the detected pseudaminic acid originated from an archaeal protein-linked glycan or if it was somehow linked to protein glycosylation. Later (Kandiba et al., 2012), 5-*N*-formyl-legionaminic acid, a legionaminic acid derivative, was shown to be the final sugar of a pentasaccharide N-linked to VP4, a glycoprotein component of *Halorubrum* pleomorphic virus 1 (HRPV-1), a haloarchaeal virus that infects *Halorubrum* sp. PV6 (Pietilä et al., 2009, 2010). As HRPV-1 does not encode components of a pathway for legionaminic acid biosynthesis, it was predicted that the sugar was in fact synthesized by a pathway found in the archaeal host strain and added to the VP4 protein during viral particle assembly (Kandiba et al., 2012).

In the present report, a set of bioinformatics, mass spectrometry (MS), and genetic and biochemical experiments provide evidence for a legionaminic acid being a component of an N-linked glycan derived from the *Halorubrum* sp. PV6 S-layer glycoprotein and that *Halorubrum* sp. PV6 encodes a legionaminic acid biosynthesis pathway. Moreover, the genes encoding the components of this pathway are seemingly part of a larger N-glycosylation gene cluster in this organism.

## Materials and Methods

### Strains and Growth Conditions

*Halorubrum* sp. PV6 cells were grown in MGM-broth containing 23% artificial salt water (3.1 M NaCl, 113 mM MgCl_2_⋅6H_2_O, 109 mM MgSO_4_.7H_2_O, 72 mM KCl, 3.8 mM CaCl_2_, 61 mM Tris–HCl, pH 7.2), 0.5% (w/v), oxoid peptone, and 0.1% (w/v) yeast extract at 37°C. *Haloferax volcanii* WR536 (H53) strain cells were grown in complete medium containing 3.4 M NaCl, 0.15 M MgSO_4_.7H_2_O, 1 mM MnCl_2_, 4 mM KCl, 3 mM CaCl_2_, 0.3% (w/v) yeast extract, 0.5% (w/v) tryptone, 50 mM Tris–HCl, and pH 7.2 (Mevarech and Werczberger, 1985) at 42°C.

### Bioinformatics

The *Halorubrum* sp. PV6 genome sequence was determined (accession number CP030064, Roine, Bamford, and Holm, manuscript in preparation), open reading frames (ORFs) were predicted using Prodigal (Hyatt et al., 2010) and RAST (Aziz et al., 2008; Overbeek et al., 2014) and automatically annotated using RAST and PANNZER 2 (Koskinen et al., 2015). For further annotation, HHpred (Söding et al., 2005) and Phyre2 (Kelley et al., 2015) were employed. The genomic region spanning the N-glycosylation gene cluster has been submitted to the GenBank nucleotide database (accession number MH673034).

Various other bioinformatics tools were used to describe the *Halorubrum* sp. PV6 S-layer glycoprotein. The signal peptide of the protein was predicted using SignalP 4.1 set for Gram-positive bacteria^1^. Alignment of the *Halorubrum* sp. PV6 and *Hfx. volcanii* S-layer glycoproteins was performed using ClustalW^2^, with shading being added using the Boxshade tool at the same site. The extent of homology between two proteins was determined using the BL2SEQ tool also found at this site.

### Cell Wall Preparation

To enrich the S-layer glycoprotein, stationary phase *Halorubrum* sp. PV6 cells were collected, washed, and resuspended in 3.4 M NaCl and 27 mM KCl and incubated with shaking at room temperature for 15 min. After centrifugation (Sorvall SA-300, 9,000 rpm, 15 min, 4°C), the supernatant was collected, MgSO_4_ was added to a final concentration of 160 mM, and incubated at 4°C overnight. S-layer glycoprotein was harvested by pelleting (Sorvall T-1270, 41,000 rpm, 20 min, 4°C). The pellet was resuspended in protein sample buffer, subjected to 12% SDS-PAGE and stained with Coomassie R-250 (Fluka).

### Liquid Chromatography–Electrospray Ionization Mass Spectrometry (LC–ESI MS)

For LC-ESI MS analysis of the *Halorubrum* sp., PV6 S-layer glycoprotein was conducted essentially as reported previously (Calo et al., 2011). In-gel digestion of the S-layer glycoprotein was first conducted. The S-layer glycoprotein-containing band was excised from the gel using a clean scalpel, destained in 400 μl of 50% (vol/vol) acetonitrile (Sigma) in 40 mM NH_4_HCO_3_, pH 8.4, dehydrated with 100% acetonitrile, and dried using a SpeedVac drying apparatus. The S-layer glycoprotein was reduced with 10 mM dithiothreitol (Sigma) in 40 mM NH_4_HCO_3_ at 56°C for 60 min and then alkylated for 45 min at room temperature with 55 mM iodoacetamide in 40 mM NH_4_HCO_3_. The gel pieces were washed with 40 mM NH_4_HCO_3_ for 15 min, dehydrated with 100% acetonitrile, and SpeedVac-dried. The gel slices were rehydrated with 12.5 ng/μl of mass spectrometry (MS) grade Trypsin Gold (Promega) in 40 mM NH_4_HCO_3_ and incubated overnight at 37°C. The protease-generated peptides were extracted with 0.1% (v/v) formic acid in 20 mM NH_4_HCO_3_, followed by sonication for 20 min at room temperature, dehydration with 50% (v/v) acetonitrile, and additional sonication. After three rounds of extraction, the gel pieces were dehydrated with 100% acetonitrile and dried completely with a SpeedVac. Next, 12.5 ng/μl Glu-C (V8) protease (Promega, sequencing-grade) in 40 mM NH_4_HCO_3_ were added. After an overnight incubation at 37°C, the sample was dried completely with a SpeedVac, resuspended in 5% (v/v) acetonitrile containing 1% formic acid (v/v) and infused into the mass spectrometer using static nanospray Econotips (New Objective, Woburn, MA, United States). The protein digests were separated online by nano-flow reverse-phase liquid chromatography (LC) by loading onto a 150-mm by 75-μm (internal diameter) by 365-μm (external diameter) Jupifer pre-packed fused silica 5-μm C_18_ 300Å reverse-phase column (Thermo Fisher Scientific, Bremen, Germany). The sample was eluted into the LTQ Orbitrap XL mass spectrometer (Thermo Fisher Scientific) using a 60-min linear gradient of 0.1% formic acid (v/v) in acetonitrile/0.1% formic acid (1:19, by volume) to 0.1% formic acid in acetonitrile/0.1% formic acid (4:1, by volume) at a flow rate of 300 nl/min.

### Reverse Transcriptase Polymer Chain Reaction (RT-PCR)

To assess the transcription and co-transcription of ORFs contributing to a putative legionaminic acid biosynthesis pathway, RT-PCR was performed. Specific forward and reverse oligonucleotide primers were designed to amplify each *Halorubrum* sp. PV6 ORF under consideration, as well as stretches beginning in a given sequence and ending in the downstream sequence (for primer sequences, see **Supplementary Tables S1** and **S2**). RNA isolation was carried out using an RNeasy mini-kit (Qiagen) according to the manufacturer’s instructions. Contaminating DNA in the RNA samples was eliminated with a RNase-Free DNase Set (Qiagen) during RNA extraction. RNA concentration was determined spectrophotometrically. Single-stranded cDNA was prepared from the extracted RNA using an Oligo(dT) primer in a Superscript IV 1st Strand System (Invitrogen). Together with appropriate forward and reverse primer pairs, the cDNA generated was used for PCR amplification. cDNA amplification was monitored by electrophoresis in 1% agarose gels. To exclude possible contributions from contaminating DNA, control experiments were conducted in which PCR amplification was performed on total RNA prior to cDNA preparation.

### Expression and Purification of *Halorubrum* sp. PV6 LegI and LegF

To generate plasmids pWL-CBD-LegI and pWL-CBD-LegF encoding *Clostridium thermocellum* cellulose-binding domain (CBD; Morag et al., 1995)-tagged *Halorubrum* sp. PV6 legionaminic acid synthase (LegI; Schoenhofen et al., 2009) and CMP-legionaminic acid synthase (LegF; Schoenhofen et al., 2009), respectively, *HrrPV6_1053* (encoding LegI) and *HrrPV6_1048* (encoding LegF) were PCR-amplified from *Halorubrum* sp. PV6 genomic DNA using primers designed to introduce *Nde*I and *Kpn*I restriction sites at the 5′ and 3′ ends of the LegI- and LegF-encoding sequences, respectively, as well as 25 bp-long overlaps with plasmid pWL-CBD (Irihimovitch and Eichler, 2003) at each end of the amplified fragments (legI forward primer: CGGTGGCAGTGTAGTAGGAGGTCATATGGAGATTGATGGTACCCG; legI reverse primer: GGCCAGCGGGAGATCCCCGGGTACCTTAATCCTCACAACCAGTACC; legF forward primer: CGGTGGCAGTGTAGTAGGAGGTCATATGTCCACCCGCACATTAGC; legF reverse primer: GGCCAGCGGGAGATCCCCGGGTACCTTAATCCTCACAACCAGTACC). The PCR products were ligated into plasmid pWL-CBD, previously digested with *Nde*I and *Kpn*I, using the Gibson Assembly Kit (New England BioLabs), according to the manufacturer’s instructions. The assembled plasmids were introduced into *Escherichia coli* Cloni cells (Lucigen) and then into *Hfx. volcanii* cells.

Cellulose-binding domain-tagged LegI and LegF were purified as previously described (Irihimovitch and Eichler, 2003). Briefly, 500 ml of *Hfx. volcanii* cells transformed to express the CBD-tagged LegI or LegF were grown to mid-logarithmic phase, harvested, and resuspended in 60 ml solubilization buffer (1% Triton X-100, 2 M NaCl, 50 mM Tris–HCl, pH 7.2) containing 400 U benzonase nuclease (Novagen) and 0.5 mM PMSF. The solubilized mixture was nutated for 20 min at room temperature and subjected to sonication (2 s on and 1 s off for 90 s, 70% output, Misonix XL2020 ultrasonicator) on ice and centrifugation (10,500 *g*, 30 min, 4°C). Cellulose (400 μl of a 10% (w/v) solution; Sigma) was added to 10 ml aliquots of the supernatant. After a 1-h nutation at 4°C, the suspensions were centrifuged (2,650 *g*, 2 min, 4°C), the supernatants were discarded and the cellulose pellets were washed four times with wash buffer containing 2 M NaCl, 50 mM Tris–HCl, pH 7.2. After the final wash, the cellulose beads were centrifuged (2,650 *g*, 2 min, 4°C), the supernatants were removed and the pellets, containing cellulose beads linked to the CBD-tagged proteins, were resuspended in SDS-PAGE sample buffer, boiled for 5 min and centrifuged (2,000 rpm, 2 min). The solubilized proteins were separated by 12% SDS-PAGE and visualized by InstantBlue (Expedeon) staining. Alternatively, 2 ml of the washed cellulose beads containing the bound CBD-tagged proteins were used for *in vitro* assaying of LegI and LegF function.

### Assaying *Halorubrum* sp. PV6 LegI Activity

*Halorubrum* sp. PV6 LegI activity was assessed by confirming the ability of the enzyme to catalyze the same reaction as does NeuB, its homolog in sialic acid biosynthesis (Vann et al., 1997; Glaze et al., 2008; Schoenhofen et al., 2009), as revealed by the thiobarbituric acid assay (Warren, 1959), given that the LegI substrate 2,4-diNac-6-deoxy mannose, is not commercially available. Briefly, reactions (50 μl) containing 12.5 mM *N*-acetylmannosamine (Sigma), 12.5 mM phosphoenolpyruvate (Sigma), 10 mM MnCl_2_, 2 M NaCl, 100 mM Tris–HCl, pH 7.2, and cellulose-bound enzyme were incubated at 37°C for 0–4 h, with aliquots being removed once an hour. The reactions were terminated by adding sodium periodate (137 μL of a 2.5 mg/ml stock solution prepared in 57 mM H_2_SO_4_; Sigma) and incubating at 37°C for 15 min. Sodium arsenite (50 μL of a 25 mg/ml stock solution prepared in 0.5 M HCl; Sigma) was added and the reactions were shaken until the brown color that developed disappeared. Next, 2-thiobarbituric acid (100 μL of a 71 mg/mL stock solution, pH 9.0 with NaOH; Alfa Aesor) was added and the mixtures were incubated at 100°C for 10 min, transferred to ice for 5 min and then held at room temperature for an additional 5 min. One milliliter of acidic butanol (*n*-butanol with 5% HCl) was added to each reaction. After shaking for 10 min, the reactions were centrifuged at 13,000 rpm for 10 min at room temperature to separate the organic and inorganic phases. The fluorescence of 100-μl aliqouts of the organic phase (containing the *N*-acetylneuraminic acid produced) was measured on a Tecan Infinite M200 plate reader (λem/ex = 585/555 nm). Fluorescence measurements were converted into *N*-acetylneuraminic acid concentrations using a calibration curve prepared with known amounts of this nonulosonic acid (Abnova).

### Assaying *Halorubrum* sp. PV6 LegF Activity

*Halorubrum* sp. PV6 LegF activity was assessed by confirming the ability of the enzyme to catalyze the same reaction as does NeuA, its homolog in sialic acid biosynthesis (Vann et al., 1987; Haft and Wessels, 1994; Glaze et al., 2008; Schoenhofen et al., 2009), given that the LegF substrate legionaminic acid, is not commercially available. The assay was performed as described (Liu et al., 2004) with several modifications. Briefly, reactions (100 μl) containing 2.8 mM *N*-acetylneuraminic acid (Acros), 5.5 mM cytidine triphosphate (CTP; Alfa Aesor), 20 mM MgCl_2_, 1.7 M NaCl, 0.2 mM dithiothreitol (Sigma), 200 mM Tris–HCl, pH 9, and cellulose-bound enzyme were incubated at 37°C for 0–2 h, with aliquots being removed every 30 min. In each aliquot, unreacted *N*-acetylneuraminic acid was reduced with 20 μl of 1.6 M sodium borohydride (Sigma) at room temperature for 15 min. The reactions were placed on ice and 20 μl of 20 N phosphoric acid were added to decompose the sodium borohydride. After standing at 0°C for 5 min, the reactions were incubated at 37°C for 10 min to cleave the phosphoester bond of the CMP-*N*-acetylneuraminic acid formed. Free *N*-acetylneuraminic acid was oxidized with 50 μl of 0.2 M sodium periodate (Sigma) at room temperature for 15 min, at which point 160 μl of 4% sodium arsenite (Sigma) in 0.5 N hydrochloric acid were added. Thiobarbituric acid (400 μl of a 0.6% solution; Alfa Aesor) in 0.5 M sodium sulfate (Acros) were added and the samples were heated in boiling water for 15 min. After the solution had cooled, 400 μl of cyclohexanone were added, and the mixture was shaken and centrifuged at 13,000 rpm for 10 min at room temperature to separate the organic and inorganic phases. The fluorescence of 100-μl aliqouts of the organic phase (containing the *N*-acetylneuraminic acid produced) was measured on a Tecan Infinite M200 plate reader (λem/ex = 585/555 nm). Fluorescence measurements were converted into *N*-acetylneuraminic acid concentrations using a calibration curve prepared with known amounts of this nonulosonic acid (Abnova).

## Results

### The *Halorubrum* sp. PV6 S-Layer Glycoprotein Is N-Glycosylated by a Legionaminic Acid-Containing Pentasaccharide

In earlier work, we reported that VP4, the major structural protein of the haloarchaeal pleomorphic virus HRPV-1, is N-glycosylated by a pentasaccharide comprising glucose, glucuronic acid, mannose, sulphated glucuronic acid, and a terminal 5-*N*-formyl-legionaminic acid residue, when directly isolated from virions produced by the host strain, *Halorubrum* sp. PV6 (Kandiba et al., 2012). The present study began with efforts to determine whether native glycoproteins of *Halorubrum* sp. PV6 are also N-glycosylated by the same glycan.

The S-layer glycoprotein has served as a useful reporter of archaeal N-glycosylation (Jarrell et al., 2010). Accordingly, N-glycosylation of the predicted *Halorubrum* sp. PV6 S-layer glycoprotein was considered here. In *Halorubrum* sp. PV6, the predicted S-layer glycoprotein (HrrPV6_1002) corresponds to an 805 residue-long protein, preceded by an apparent 31 residue-long signal peptide. Alignment of the amino acid sequence of the predicted *Halorubrum* sp. PV6 S-layer glycoprotein with its *Hfx. volcanii* counterpart (HVO_2072) revealed that the two share 40% identity and 59% similarity (*E* value = 4e-169; score = 579 bits over the entire sequence length; **Supplementary Figure 1**). Both proteins present seven putative N-glycosylation sites, namely Asn-94, Asn-138, Asn-163, Asn-267, Asn-273, Asn-315 and Asn-357 in the *Halorubrum* sp. PV6 protein and Asn-13, Asn-83, Asn-274, Asn-279, Asn-370, Asn-498, and Asn-732 in the *Hfx. volcanii* protein. In the latter, several of these sites have been experimentally confirmed as being modified (Mengele and Sumper, 1992; Abu-Qarn et al., 2007; Kaminski et al., 2013b; Parente et al., 2014; Kandiba et al., 2016). The alignment further revealed that several of these putative or known N-glycosylation sites coincide or are found in close proximity to each other in the two proteins. Both S-layer glycoproteins also present a string of O-glycosylation targets near their C-terminus. In the *Hfx. volcanii* protein, an unspecified number of these Thr residues are decorated with a disaccharide comprising glucose and galactose (Sumper et al., 1990). Finally, both proteins share a C-terminal region that includes a transmembrane domain and a motif putatively recognized by ArtA, the archaeal archaeosortase involved in replacing this domain with a lipid anchor (Abdul Halim et al., 2013; Kandiba et al., 2013).

**FIGURE 1:**
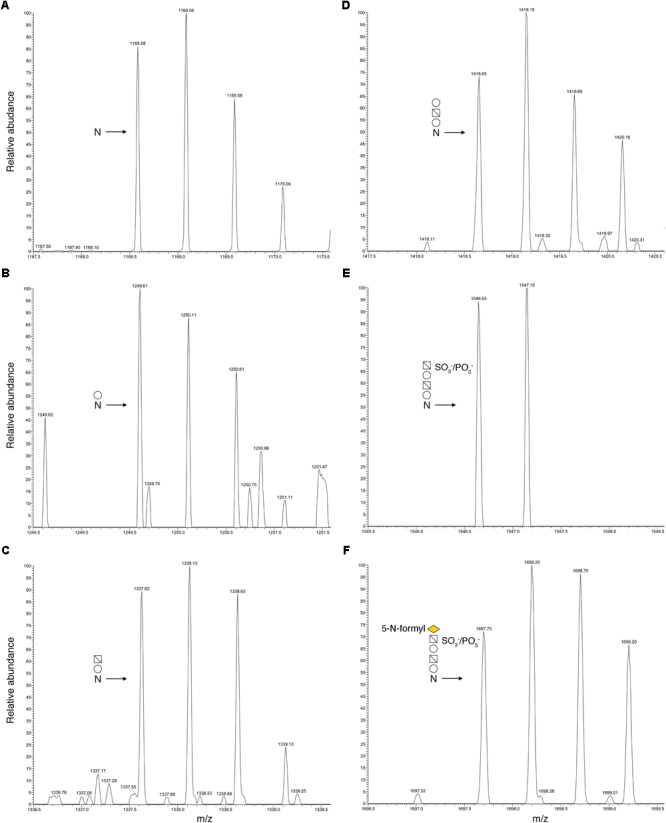
N-glycosylation profile of *Halorubrum* sp. PV6 S-layer glycoprotein Asn-94. LC-ESI/MS analysis of the Asn-94-containing peptide derived from the *Halorubrum* sp. PV6 S-layer glycoprotein following digestion with trypsin and GluC protease was performed. Shown are doubly charged [M+2H]^2+^ ion peaks corresponding to **(A)** the ^78^TGSYAIGGPDAADGAFNVTVVTPR^101^ peptide (*m/z* 1168.58), and the same peptide successively modified by **(B)** a hexose (*m/z* 1249.61), **(C)** a hexuronic acid (*m/z* 1337.62), **(D)** a hexose (*m/z* 1418.65), **(E)** a sulfated/phosphorylated hexuronic acid (*m/z* 1546.65), and **(F)** a 5-*N*-formyl-legionaminic acid (*m/z* 1697.70). In each panel, the N-glycosylation status of the peptide is schematically depicted, where “*N*” corresponds to Asn-94. Employing symbol nomenclature for glycans guidelines (Varki et al., 2015), open circles correspond to hexoses, open squares containing a diagonal correspond to hexuronic acids, and the yellow diamond corresponds to 5-*N*-formyl-legionaminic acid.

**FIGURE 2:**
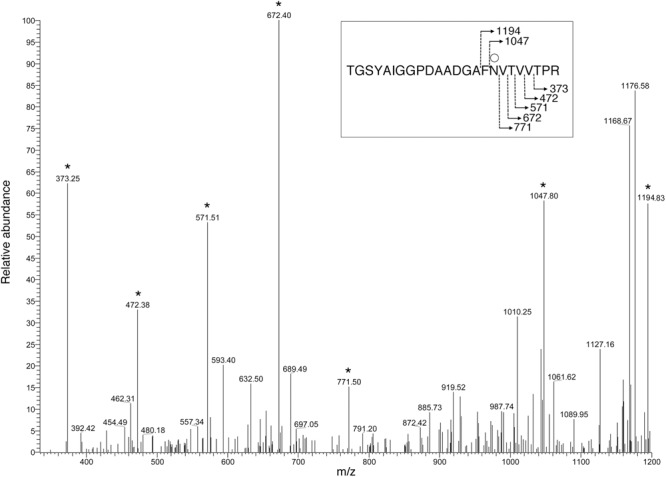
MS/MS verification of *Halorubrum* sp. PV6 S-layer glycoprotein Asn-94 glycosylation. To verify that glycosylation of the hexose-modified S-layer glycoprotein-derived peptide, ^78^TGSYAIGGPDAADGAFNVTVVTPR^101^, occurs on Asn-94, the MS spectrum of the [M+2H]^2+^ base peak of this peptide, observed at *m/z* 1249.61, was obtained *via* mass-dependent acquisition. The y-ion series includes fragments that contain the hexose-modified Asn-94 residue. The inset shows the fragmentation scheme, with the Asn94-bound hexose being represented by the open circle. Peaks corresponding to y-ion series fragments are indicated by stars.

To determine whether the predicted *Halorubrum* sp. PV6 S-layer glycoprotein is modified by the same N-linked glycan as is the virus HRPV-1 VP4 glycoprotein when synthesized in *Halorubrum* sp. PV6 (Kandiba et al., 2012), proteolytic fragments of the predicted S-layer glycoprotein were examined by LC-ESI MS. Such analysis revealed a peak of *m/z* 1168.58 (**Figure 1A**), corresponding to the [M+2H]^2+^ ion of the predicted S-layer glycoprotein-derived peptide ^78^TGSYAIGGPDAADGAFNVTVVTPR^101^ (calculated mass, *m/z* 1168.58), containing the putative N-glycosylation site Asn-94. Peaks of *m/z* 1249.61, 1337.62, 1418.65, 1546.65, and 1697.70 were also detected, consistent with calculated masses of the same Asn-94-containing peptide modified by a hexose (calculated mass *m/z* 1249.58; **Figure 1B**); a hexose and a hexuronic acid (calculated mass *m/z* 1337.58; **Figure 1C**); a hexose, a hexuronic acid, and a hexose (*m/z* 1418.58; **Figure 1D**); a hexose, a hexuronic acid, a hexose, and a sulphated/phosphorylated hexuronic acid (*m/z* 1546.58; **Figure 1E**); and a hexose, a hexuronic acid, a hexose, a sulphated/phosphorylated hexuronic acid, and 5-*N*-formyl-legionaminic acid (*m/z* 1697.58; **Figure 1F**). Glycosylation of Asn-94 was verified by MS/MS analysis of the [M+2H]^2+^ base peak of the same predicted S-layer glycoprotein-derived peptide modified by a hexose observed at *m/z* 1249.61. The product ion spectrum contains a series of y-ion fragments that confirmed the presence of a hexose attached at the Asn-94 position (**Figure 2**).

Based on these MS results, combined with those of earlier analyses (Kandiba et al., 2012), it can be concluded that the predicted *Halorubrum* sp. PV6 S-layer glycoprotein is indeed modified by the same glycan as is N-linked to the viral VP4 glycoprotein expressed in this strain. As such, this represents the first example of a NulO sugar, and in particular, a legionaminic acid, as a component of an N-linked glycan decorating a native archaeal glycoprotein.

### *Halorubrum* sp. PV6 Encodes Components of a Legionaminic Acid Biosynthesis Pathway

**FIGURE 3:**
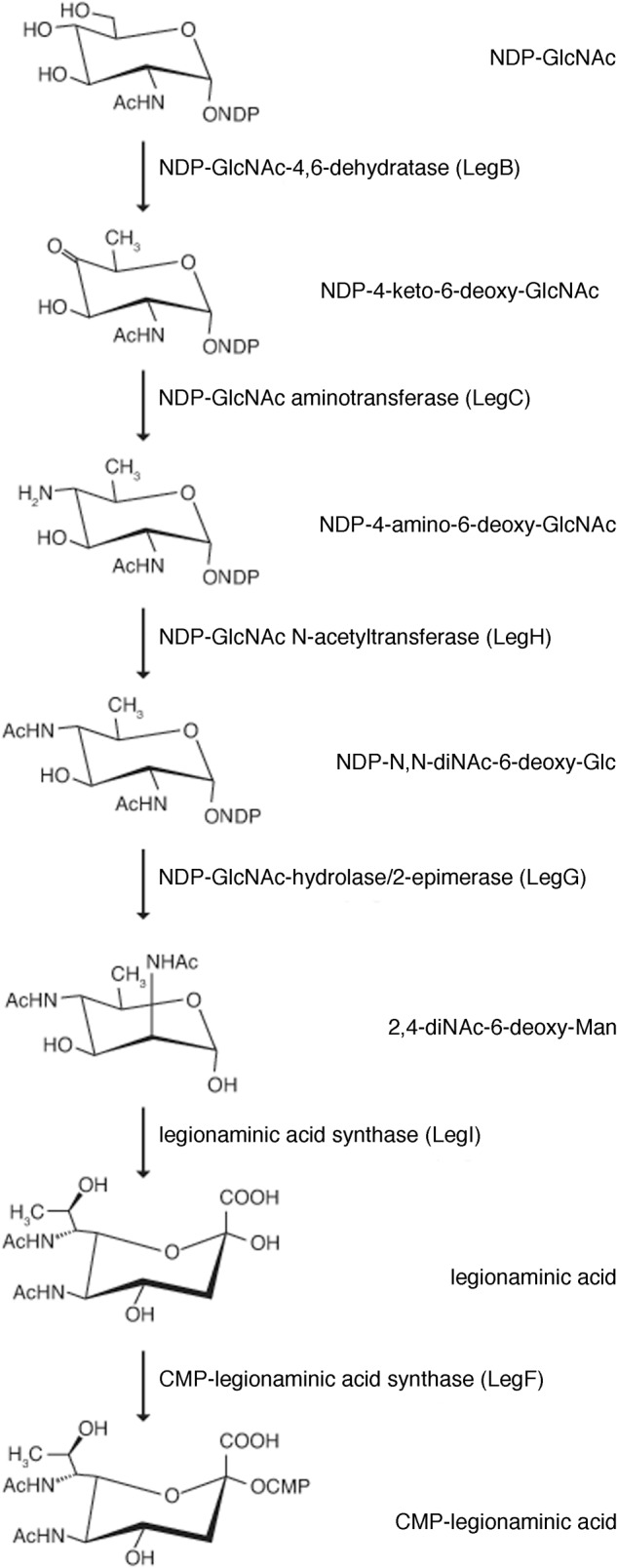
The predicted pathway of *Halorubrum* sp. PV6 legionaminic acid biosynthesis. The pathway (Glaze et al., 2008; Schoenhofen et al., 2009) starts with the dehydratase LegB that generates NDP-4-keto-6-deoxy-*N*-acetylglucosamine (GlcNAc) from NDP-GlcNAc. The aminotransferase LegC next produces the amino sugar NDP-4-amino-6-deoxy-GlcNAc. The acetyltransferase LegH subsequently generates NDP-*N,N*-diacetamido-6-deoxy-glucose (NDP-diacetamido-basillosamine), which is then converted into 2,4-diacetamido-6-deoxy-mannose by the hydrolyzing 2-epimerase LegG. The legionaminic synthase LegI now condenses this sugar with pyruvate to yield legionaminic acid. Finally, the sugar is activated by the actions of LegF, the CMP-legionaminic acid synthase. In the diagram, the enzymes that catalyze each pathway step, as well as the products formed, are listed.

**FIGURE 4:**
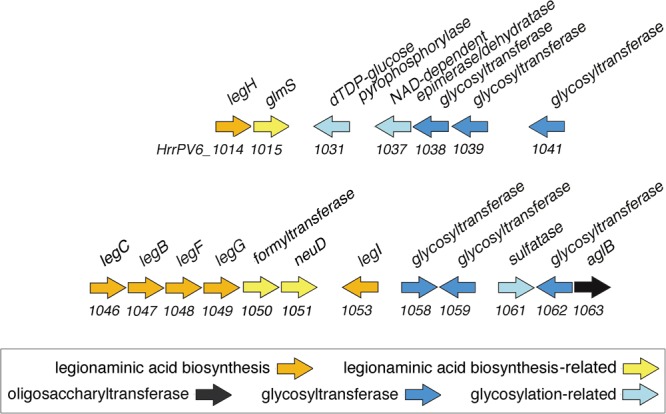
Schematic depiction of the region of the *Halorubrum* sp. PV6 genome containing ORFs encoding putative components involved in 5-*N*-formyl-legionaminic acid biosynthesis and N-glycosylation. The scheme presents the orientation but not the actual length of each ORF. Sequence information can be found at GenBank accession number MH673034.

As the predicted *Halorubrum* sp. PV6 S-layer glycoprotein is seemingly modified by an N-linked glycan that includes 5-*N*-formyl-legionaminic acid, the genomic sequence of this organism (GenBank accession number CP030064; Roine, Bamford, and Holm, manuscript in preparation) was scanned for ORFs corresponding to genes encoding components of a legionaminic acid biosynthetic pathway (**Figure 3**). Accordingly, the components of the two known pathways of legionaminic acid biosynthesis in Bacteria, namely, that of *L. pneumophila*, where UDP-conjugated intermediates are generated (Glaze et al., 2008), and that of *Campylobacter jejuni*, where GDP-conjugated intermediates are generated (Schoenhofen et al., 2009), served as queries in BLAST searches of the *Halorubrum* sp. PV6 genome. Such analysis identified sequences (see **Supplementary Material**) putatively encoding components of a *Halorubrum* sp. PV6 legionaminic acid biosynthetic pathway comprising NDP-*N*-acetylglucosamine (GlcNAc)-4,6-dehydratase (LegB; HrrPV6_1047), NDP-GlcNAc aminotransferase (LegC; HrrPV6_1046), NDP-GlcNAc *N*-acetyltransferase (LegH; HrrPV6_1014), NDP-GlcNAc-hydrolase/2-epimerase [LegG (a NeuC homolog); HrrPV6_1049], legionaminic acid synthase [LegI (a NeuB homolog); HrrPV6_1053], and CMP-legionaminic acid synthase [LegF (a NeuA homolog); HrrPV6_1048]. A schematic depiction of the region of the *Halorubrum* sp. PV6 containing these genes is presented in **Figure 4**, while information on the ORFs predicted to catalyze steps of the putative *Halorubrum* sp. PV6 legionaminic acid biosynthesis pathway is provided in **Table 1**.

**Table 1:** Sequences putatively comprising the *Halorubrum* sp. PV6 legionaminic acid biosynthesis pathway.

Sequence	Current annotation	Revised annotation
HrrPV6_1014	Nucleotidyltransferase	LegH (NDP-GlcNAc *N*-acetyltransferase)
HrrPV6_1046	PLP-dependent aminotransferase	LegC (NDP-GlcNAc aminotransferase)
HrrPV6_1047	Nucleotide sugar epimerase/dehydratase	LegB (NDP-GlcNAc-4,6-dehydratase)
HrrPV6_1048	*N*-acetylneuraminate cytidylyltransferase (NeuA)	LegF (CMP-legionaminic acid synthase)
HrrPV6_1049	UDP-*N*-GlcNAc- 2-epimerase (NeuC)	LegG (NDP-GlcNAc-hydrolase/2-epimerase)
HrrPV6_1053	*N*-acetylneuraminate synthase (NeuB)	LegI (legionaminic acid synthase)

This region of the genome also includes additional sequences encoding enzymes possibly related to 5-*N*-formyl legionaminic acid biosynthesis, such as *HrrPV6_1051*, encoding a homolog of the O-acetyltransferase NeuD involved in sialic acid biogenesis in *E. coli* (Daines et al., 2000). *HrrPV6_1050* possibly encodes the *N*-formyltransferase involved in modifying legionaminic acid. Finally, *HrrPV6_1015* is predicted to encode the glucosamine-6-phosphate synthase GlmS.

To determine whether the Leg pathway is widely distributed in the genus *Halorubrum*, the different Leg protein sequences identified here were used as queries in a series of BLAST searches of completed genome sequences of other *Halorubrum* species. This revealed the presence of the complete set of Leg proteins in only two species, namely, *Halorubrum ezzemoulense* and *Halorubrum sodomense*. This suggests that legionaminic acid is not widely produced in this genus, in agreement with earlier efforts showing the limited distribution of NulO biosynthesis pathway genes in Archaea (Kandiba and Eichler, 2013). Future efforts will, however, be needed to confirm that *Hrr. ezzemoulense* and *Hrr. sodomense* indeed synthesize and employ legionaminic acids in N-glycosylation.

### Several Genes Involved in Legionaminic Acid Biosynthesis Are Co-transcribed

**FIGURE 5:**
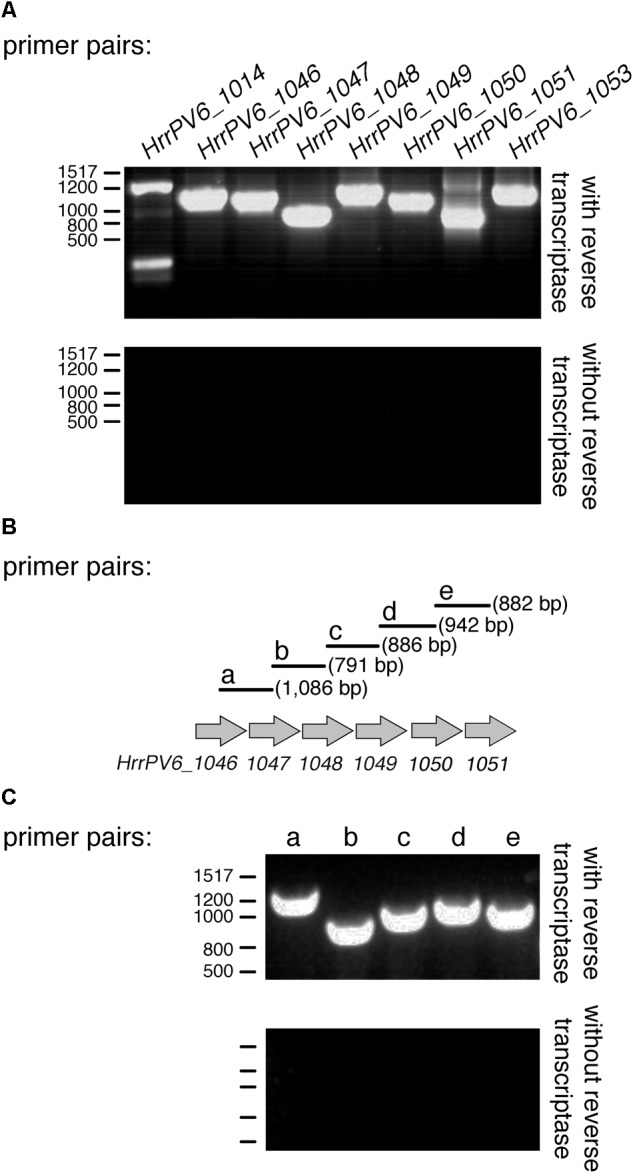
Genes putatively involved in legionaminic acid biosynthesis are co-transcribed. **(A)** RT-PCR products obtained using primer pairs designed to amplify the indicated ORF and cDNA (with reverse transcriptase; upper panel) or RNA pre-treated with DNase but not with reverse transcriptase (without reverse transcriptase; lower panel) as template. **(B)** Schematic depiction of the *Halorubrum* sp. PV6 genome containing genes encoding putative components involved in legionaminic acid biosynthesis suspected of being co-transcribed. Primer pairs beginning within the upstream sequence and ending within the downstream sequence are schematically depicted. Primer pairs a, b, c, d, and e are designed to amplify products of the listed sizes. **(C)** RT-PCR products obtained using each primer pair and cDNA (with reverse transcriptase; upper panel) or RNA pre-treated with DNase but not with reverse transcriptase (without reverse transcriptase; lower panel) as template. In **(A,C)**, the positions of bp markers are provided on the left of each panel and values are listed on the left of the upper profile.

Because transcription of an ORF is a good indicator of that sequence being a true protein-encoding gene, the generation of RNA from the sequences corresponding to *legH* (*HrrPV6_1014*), *legC* (*HrrPV6_1046*), *legB* (*HrrPV6_1047*), *legF* (*HrrPV6_1048*), *legG* (*HrrPV6_1049*), a predicted formyltransferase-encoding gene (*HrrPV6_1050*), *neuD* (*HrrPV6_1051*), and *legI* (*HrrPV6_1053*) was considered by RT-PCR. Accordingly, cDNA prepared from RNA isolated from exponentially growing *Halorubrum* sp. PV6 cells using reverse transcriptase served as template in a series of PCR amplifications involving primers designed to generate PCR products corresponding to each ORF. In each case, products of the expected lengths (*HrrPV6_1014*: 1,368 bp; *HrrPV6_1046*: 1,139 bp; *HrrPV6_1047*: 1,025 bp; *HrrPV6_1048*: 722 bp; *HrrPV6_1049*: 1,163 bp; *HrrPV6_1050*: 953 bp; *HrrPV6_1051*: 629 bp; and *HrrPV6_1053*: 1,053 bp) were obtained (**Figure 5A**, upper panel). As negative controls, the same PCR amplifications were repeated, this time using DNase-treated RNA as template (i.e., without reverse transcriptase treatment). Now, no PCR products were obtained (**Figure 5A**, lower panel).

Examination of the region of the *Halorubrum* sp. PV6 genome that spans *HrrPV6_1046* to *HrrPV6_1051* revealed these genes to be not only adjacent but also to be similarly oriented. As such, the possibility that several or all of these genes are co-transcribed was next considered by RT-PCR in a series of amplifications involving primers designed to generate PCR products spanning regions within and between adjacent ORFs (**Figure 5B**). When PCR was performed with these primers pairs and the cDNA template, products of the expected sizes were obtained in each case (**Figure 5C**, upper panel). As negative controls, the same PCR amplifications were repeated, this time using extracted RNA pre-treated with DNase but not with reverse transcriptase as template. Now, no PCR products were obtained (**Figure 5C**, lower panel).

It can thus be concluded that *HrrPV6_1046, HrrPV6_1047, HrrPV6_1048, HrrPV6_1049, HrrPV6_1050, HrrPV6_1051*, and *HrrPV6_1053* correspond to protein-encoding genes and, moreover, that *HrrPV6_1046, HrrPV6_1047, HrrPV6_1048, HrrPV6_1049, HrrPV6_1050*, and *HrrPV6_1051* are co-transcribed.

### Biochemical Characterization of *Halorubrum* sp. PV6 LegI and LegF

**FIGURE 6:**
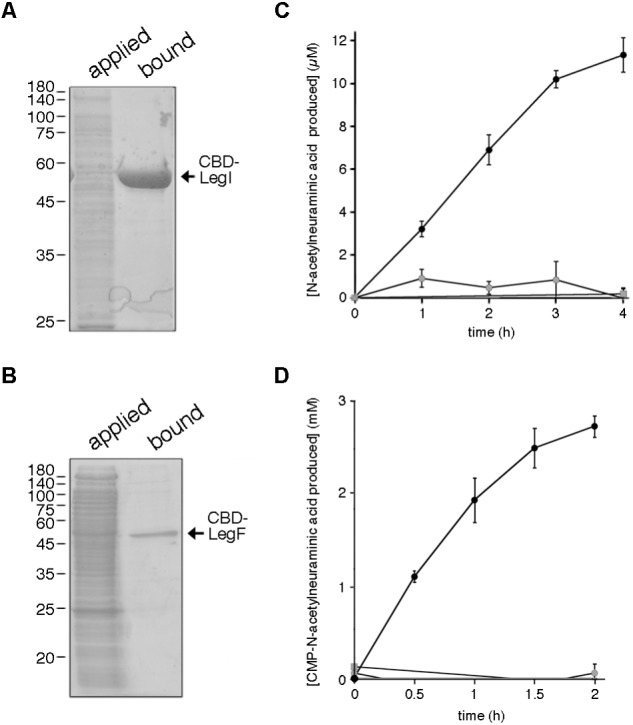
*Halorubrum* sp. PV6 LegI and LegF contribute to legionaminic acid biosynthesis. **(A)** *Halorubrum* sp. PV6 LegI and **(B)** LegF were purified from total lysates of *Hfx. volcanii* expressing each CBD-tagged protein (applied) on cellulose (bound). Aliquots of each pool were separated by 12% SDS-PAGE and Coomassie stained. In each panel, the positions of molecular weight markers are indicated on the left, while the position of the purified protein is indicated on the right. **(C)** LegI activity was confirmed in reactions containing cellulose-bound CBD-LegI, *N*-acetylmannosamine, and phosphoenolpyruvate (black circles) or in which the *N*-acetylmannosamine (gray circles) or phosphoenolpyruvate (gray squares) were omitted, or where cellulose incubated with the lysate of *Hfx. volcanii* not expressing the CBD-tagged protein was added instead of CBD-LegI-bearing beads (gray triangles). Each point is the average of three repeats ± SEM. **(D)** LegF activity was confirmed in reactions containing cellulose-bound CBD-LegF, *N*-acetylneuraminic acid and CTP (black circles) or in which the *N*-acetylneuraminic acid (gray circles) or CTP (gray squares) were omitted, or where cellulose incubated with the lysate of *Hfx. volcanii* not expressing the CBD-tagged protein was added instead of CBD-LegF-bearing beads (gray triangles). The amount of *N*-acetylneuraminic acid produced in each reaction was determined every 30 min over a 2 h interval, except in the latter two controls, when measurements were only taken at the start and the end of the experiment. Each point is the average of three repeats ± SEM.

To verify the actions of proteins assigned to the predicted *Halorubrum* sp. PV6 legionaminic acid biosynthesis pathway, biochemical studies were undertaken. *Hfx. volcanii* cells were transformed to express the predicted legionaminic acid synthase (LegI) bearing an N-terminal *C. thermocellum* CBD tag. *Hfx. volcanii* was used as a heterologous expression platform in these experiments since no genetic tools presently exist for the manipulation of *Halorubrum* sp. PV6. The presence of this CBD tag allows for cellulose-based purification in the presence of molar concentrations of salt, as required for the proper folding and activity of haloarchaeal enzymes (Irihimovitch and Eichler, 2003). Cellulose-based purification of CBD-tagged LegI (59.5 kDa) and LegF (45.3 kDa) from a total cell lysate of *Hfx. volcanii* cells transformed to express each protein is presented in **Figures 6A,B**, respectively.

In legionaminic acid biosynthesis, LegI catalyzes the condensation of 2,4-di-*N*-acetyl-6-deoxymannose and phosphoenolpyruvate to generate legionaminic acid (Glaze et al., 2008; Schoenhofen et al., 2009). As this is essentially the same reaction as catalyzed by the LegI homolog NeuB (sialic acid synthase) during sialic acid biosynthesis (Vann et al., 1997), the ability of cellulose-bound CBD-tagged *Halorubrum* sp. PV6 LegI to condense *N*-acetylmannosamine and phosphoenolpyruvate, yielding *N*-acetylneuraminic acid, was determined. As reflected in **Figure 6C**, incubation of cellulose-bound LegI with the two substrates led to a linear increase in sialic acid levels over the first 3 h, activity which began to taper off during the fourth hour of incubation. By contrast, no *N*-acetylneuraminic acid was generated when either *N*-acetylmannosamine or phosphoenolpyruvate were omitted from the reaction, or when cellulose beads pre-incubated with a lysate prepared from *Hfx. volcanii* cells not transformed to express CBD-tagged LegI were tested.

The conversion of legionaminic acid to CMP-legionaminic acid by *Halorubrum* sp. PV6 LegF (Glaze et al., 2008; Schoenhofen et al., 2009) was next considered by addressing the ability of the enzyme to catalyze the parallel NeuA-mediated reaction that occurs in sialic acid biogenesis (Vann et al., 1987). Accordingly, the ability of cellulose-bound CBD-tagged LegF to generate CMP-*N*-acetylneuraminic acid from *N*-acetylneuraminic acid and CTP was determined. Incubation of cellulose-bound LegF with the two substrates led to a constant increase in CMP-*N*-acetylneuraminic acid levels that appeared to approach saturation over the course of the experiment (**Figure 6D**). By contrast, no *N*-acetylneuraminic acid was generated when either *N*-acetylneuraminic acid or CTP were omitted from the reaction, or when cellulose beads pre-incubated with a lysate prepared from *Hfx. volcanii* cells not transformed to express CBD-tagged LegF were tested.

### Putative *Halorubrum* sp. PV6 N-Glycosylation Pathway Components

In seeking ORFs putatively encoding components of a legionaminic acid biosynthesis pathway in *Halorubrum* sp. PV6, it was noted that the predicted product of *HrrPV6_1063* is annotated as an oligosaccharyltransferase. Indeed, HrrPV6_1063 shares 46% identity and 59% similarity (87% coverage; score 757 bits) with *Hfx. volcanii* AglB, the confirmed oligosaccharyltransferase in this organism (Abu-Qarn et al., 2007). As such, additional putative N-glycosylation pathway component-encoding sequences were sought in the neighboring ORFs, given how in halophiles, as in certain other Archaea, genes encoding N-glycosylation pathway components tend to cluster around *aglB* (Magidovich and Eichler, 2009; Kaminski et al., 2013a; Kandiba and Eichler, 2015). In this manner, *Halorubrum* sp. PV6 ORFs predicted to encode glycosyltransferases and other enzymes putatively involved in N-linked glycan biosynthesis and assembly were identified in the immediate and near vicinity of the putative AglB encoding sequence (**Table 2**). It is reasonable to assume that these sequences, together with those sequences encoding the legionaminic acid biosynthesis pathway, comprise the bulk of the *Halorubrum* sp. PV6 N-glycosylation pathway (**Figure 4**). That portion of the *Halorubrum* sp. PV6 genome that contains genes encoding N-glycosylation pathway components, as well as genes encoding the legionaminic acid biosynthesis pathway, have been submitted to GenBank and assigned accession number MH673034.

**Table 2:** Sequences putatively encoding components of a *Halorubrum* sp. PV6 N-glycosylation pathway.

Sequence	Current annotation
HrrPV6_1031	dTDP-glucose pyrophosphorylase
HrrPV6_1037	NAD-dependent epimerase/hydratase
HrrPV6_1038	Glycosyltransferase
HrrPV6_1039	Glycosyltransferase
HrrPV6_1041	Glycosyltransferase
HrrPV6_1058	Glycosyltransferase
HrrPV6_1059	Glycosyltransferase
HrrPV6_1061	Sulfatase
HrrPV6_1062	Glycosyltransferase
HrrPV6_1063	Oligosaccharyltransferase (AglB)

## Discussion

Although currently limited in numeric terms, those N-linked glycans decorating archaeal glycoproteins for which compositional and/or structural data is available reveal a degree of diversity not seen in their eukaryotic or bacterial counterparts (Schwarz and Aebi, 2011; Eichler, 2013). The list of sugars found as part of N-linked glycans in Archaea has been expanded to include 5-*N*-formyl-legionaminic acid, first detected in the glycan of a major structural protein of a virus that infects *Halorubrum* sp. PV6 (Kandiba et al., 2012) and now inferred to be the terminal sugar of the glycan N-linked the S-layer glycoprotein in this haloarchaea. The present report, moreover, delineated a pathway for legionaminic acid biosynthesis in *Halorubrum* sp. PV6, demonstrated the transcription of pathway genes (as well as the co-transcription of several of these genes) and confirmed the enzymatic activities of two pathway components, LegI and LegF. Finally, the delineation of a pathway for legionaminic acid biosynthesis in *Halorubrum* sp. PV6 implies that Archaea are capable of generating NDP-di-*N*-acetylbacillosamine (2,4-diacetamido-2,4,6-trideoxy-D-glucose) through the actions of LegH. UDP-di-*N*-acetylbacillosamine is important in bacterial protein glycosylation, providing the first sugar of both the O-linked glycan decorating glycoproteins in *Neisseria gonorrhoeae* (Aas et al., 2007; Hartley et al., 2011) and the N-linked glycan in *C. jejuni* (Young et al., 2002; Glover et al., 2006).

In *Halorubrum* sp. PV6, the components of a predicted legionaminic acid biosynthesis pathway share a common region spanning some 67 kB of the genome with ORFs predicted to encode enzymes that participate in the biogenesis of the remaining sugars of the N-linked pentasaccharide decorating the S-layer glycoprotein. These could include the legionaminic acid transferase that has yet to be identified in any organism employing this sugar in protein glycosylation. However, because no genetic tools yet exist for the manipulation of *Halorubrum* sp. PV6, it is not yet possible to rely on gene deletion to directly test the involvement of any these components in either legionaminic acid biosynthesis or N-glycosylation. Instead, sequences of interest can be expressed in other halophilic archaea for which appropriate molecular techniques, such as transformation, exist, including *Hfx. volcanii*. Indeed, *Halorubrum* sp. PV6 LegF and LegI characterized here were expressed in *Hfx. volcanii*. For those ORFs encoding proteins that are assigned various roles in *Halorubrum* sp. PV6 N-glycosylation, studies in which the abilities of these sequences to complement *Hfx. volcanii* strains deleted of the homologous sequence, thereby restoring the missing activity, are ongoing. This approach has been previously used to confirm the actions of oligosaccharyltransferase-encoding *aglB* sequences from *Haloarcula marismortui, Halobacterium salinarum*, and *Haloferax mediterranei* (Cohen-Rosenzweig et al., 2014) and to identify other components of a *Hbt. salinarum* N-glycosylation pathway (Kandiba and Eichler, 2015).

While various functions have been assigned to the NulO sugars found as components of N-linked glycans in Eukarya and Bacteria (Kelm and Schauer, 1997; Schauer, 1982; Chen and Varki, 2010; Varki and Gagneux, 2012; Morrison and Imperiali, 2014; Schnaar et al., 2014; Varki et al., 2017), the roles served by these sugars in Archaea remains an open question, given the overall lack of information on NulO sugars as components of the N-linked glycans that decorate archaeal glycoproteins. At the same time, previous reports provided evidence for 5-*N*-formyl-legionaminic acid as being involved in the interaction between *Halorubrum* sp. PV6 and HRPV-1 that infects these cells (Kandiba et al., 2012). The HRPV-1 VP4 spike protein that protrudes from the viral membrane surface and which is thought to mediate host recognition and the initial stages of infection is modified by the same N-linked glycan that is apparently bound to the *Halorubrum* sp. PV6 S-layer glycoprotein (Pietilä et al., 2009; Kandiba et al., 2012). Viral adsorption, as reflected in the level of virus production in infected cells, was reduced in the presence of the sialic acid *N*-acetylneuraminic acid. The added sialic acid likely competed with the virus for a binding site on the *Halorubrum* sp. PV6 host that recognizes the 5-*N*-formyl-legionaminic acid that caps the N-linked glycan decorating both the S-layer glycoprotein and VP4. This raises questions as to the intended role played by this binding site in *Halorubrum* sp. PV6. Recently, glycosylation of the S-layer glycoprotein was shown to be important for cell-cell recognition and mating in *H. volcanii* (Shalev et al., 2017). It is tempting to speculate that *Halorubrum* sp. PV6 relies on the 5-*N*-formyl-legionaminic acid unit of the S-layer glycoprotein N-linked pentasaccharide for a similar purpose.

In summary, the findings presented here further expand the growing list of sugars employed in the archaeal version of N-glycosylation and provides new information on how one such sugar, 5-*N*-formyl-legionaminic acid, is generated. Such knowledge could help provide novel insight into the importance of this sugar, and indeed, roles played by N-glycosylation in archaea.

## Author Contributions

ER and JE conceived the study. MZ performed the experiments. MZ, ER, and JE analyzed the data. JE wrote the manuscript. All authors read and commented on the manuscript.

## Conflict of Interest Statement

The authors declare that the research was conducted in the absence of any commercial or financial relationships that could be construed as a potential conflict of interest.

## Footnotes

1 http://www.cbs.dtu.dk/services/SignalP/

2 https://www.expasy.org/genomics/sequence_alignment
