# Supplementary material for: Sialic Acid-Like Sugars in Archaea: Legionaminic Acid Biosynthesis in the Halophile Halorubrum sp. PV6
Source: Front Microbiol. 2018 Sep 7;9:2133. doi: 10.3389/fmicb.2018.02133 (PMC6137143; doi:10.3389/fmicb.2018.02133)
Supplement: Supplementary file 1 [file Table_1.DOCX]

**Supplementary Table S1 – Primers used for RT-PCR amplification of ORFs**

| **Primer pair** | **Primer**  **direction** | **Primer sequence** |
| --- | --- | --- |
| 1014 | Forward | ATGAACGACGAGCCC |
|  | Reverse | TCAGTCCGCTTTCCGC |
| 1046 | Forward | ATGACCCGGAAACCAGACTC |
|  | Reverse | CTGATCCACCGCTTCGATC |
| 1047 | Forward | ATGACATCCCTCGAAGGAAAAG |
|  | Reverse | TGTCTCGGGAATGTCTTCG |
| 1048 | Forward | ATGTCCACCCGCACATTAGC |
|  | Reverse | TTAATCCTCACAACCAGTACCATTC |
| 1049 | Forward | ATGACCACTGATACTCGAAATG |
|  | Reverse | ATAGGTCAGGCGTTTTCGGAT |
| 1050 | Forward | ATGCGAGTCGTGTTTATCA |
|  | Reverse | TTTGGTCTCAAGTAGAAAGTC |
| 1051 | Forward | ATGAAAGTAATTTACTGTGCTGGGG |
|  | Reverse | TTCAGTCTCAGTTGGTGAC |
| 1053 | Forward | GAGATTGATGGTACCCG |
|  | Reverse | AAACCACCTTCCTCTATCTCCA |
