# Supplementary material for: Sialic Acid-Like Sugars in Archaea: Legionaminic Acid Biosynthesis in the Halophile Halorubrum sp. PV6
Source: Front Microbiol. 2018 Sep 7;9:2133. doi: 10.3389/fmicb.2018.02133 (PMC6137143; doi:10.3389/fmicb.2018.02133)
Supplement: Supplementary file 2 [file Table_2.DOCX]

**Supplementary Table S2 – Primers used in RT-PCR co-transcription studies**

| **Primer pair** | **Primer direction and position** | **Primer sequence** |
| --- | --- | --- |
| a | Forward, in *HrrPV6_1046* | AATCGGAACCAATATTCGAATGTC |
|  | Reverse, in *HrrPV6_1047* | TCCGGATCAGTTACCGTTACTG |
| b | Forward, in *HrrPV6_1047* | GTTCTCAAGATGCCAACCTTCC |
|  | Reverse, in *HrrPV6_1048* | ACTGGGGAGGAGTAACGTATTC |
| c | Forward, in *HrrPV6_1048* | GAATACGTTACTCCTCCCCAGT |
|  | Reverse, in *HrrPV6_1049* | CTCCAGCAAGAATGTCATCG |
| d | Forward, in *HrrPV6_1049* | AATGGGTGAAGAAGAGTGGCG |
|  | Reverse, in *HrrPV6_1050* | ACGAATAGAAGATCTGGTTCATAGTCC |
| e | Forward, in *HrrPV6_1050* | GATACGTGGACAATCGAGACCTTAC |
|  | Reverse, in *HrrPV6_1051* | GATACGTGGACAATCGAGACCTTAC |
