# Supplementary material for: Sialic Acid-Like Sugars in Archaea: Legionaminic Acid Biosynthesis in the Halophile Halorubrum sp. PV6
Source: Front Microbiol. 2018 Sep 7;9:2133. doi: 10.3389/fmicb.2018.02133 (PMC6137143; doi:10.3389/fmicb.2018.02133)
Supplement: Supplementary file 3 [file Image_1.PDF]

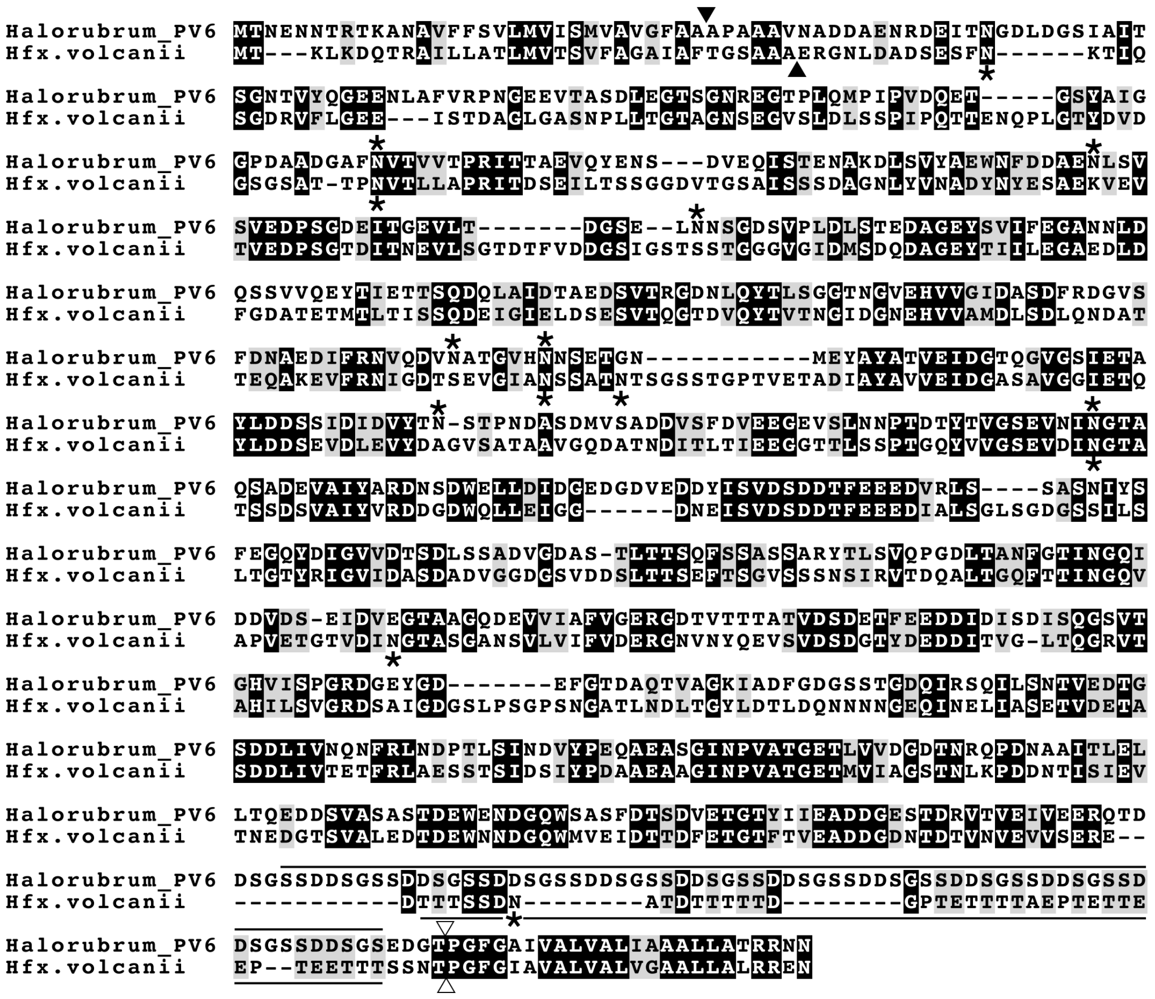

Supplementary Fig. S1 – Alignment and comparison of the *Halorubrum* sp. PV6 and *Hfx. volcanii* S-layer glycoproteins. The amino acid sequences of the *Halorubrum* sp. PV6 and *Hfx. volcanii* S-layer glycoproteins (top and bottom sequences, respectively) were aligned using ClustalW. Identical residues are highlighted against a black background while similar residues are highlighted against a grey background. Asterisks above and below each sequence indicate putative sites of N-glycosylation in the corresponding sequence, the full triangles indicate predicted (*Halorubrum* sp. PV6) or known (*Hfx. volcanii*) sites of signal peptide cleavage, and the open triangle indicate the predicted (*Halorubrum* sp. PV6) or known (*Hfx. volcanii*) sites of ArtA-mediated processing. Finally, the C-terminal string of Ser (*Halorubrum* sp. PV6) or Thr (*Hfx. volcanii*) where O-glycosylation can occur are denoted by lines above or below the sequence, respectively.
